# Lessons Learned in 11 Years of Experience With Open Abdomen Treatment With Negative-Pressure Therapy for Various Abdominal Emergencies

**DOI:** 10.3389/fsurg.2021.632929

**Published:** 2021-06-04

**Authors:** Elisabeth Gasser, Daniel Rezaie, Johanna Gius, Andreas Lorenz, Philipp Gehwolf, Alexander Perathoner, Dietmar Öfner, Reinhold Kafka-Ritsch

**Affiliations:** Medical University Innsbruck, Innsbruck, Austria

**Keywords:** negative-pressure therapy, open abdomen, damage control surgery, abdominal sepsis, second look exploration

## Abstract

**Introduction:** Open abdomen (OA) treatment with negative-pressure therapy (NPT) was initiated for perforated diverticulitis and subsequently extended to other abdominal emergencies. The aim of this retrospective study was to analyze the indications, procedures, duration of NPT, and the outcomes of all our patients.

**Methods:** All consecutive patients treated with intra-abdominal NPT from January 1, 2008 to December 31, 2018 were retrospectively analyzed.

**Results:** A total of 438 patients (44% females) with a median (range) age of 66 (12–94) years, BMI of 25 (14–48) kg/m^2^, and ASA class I, II, III, and IV scores of 36 (13%), 239 (55%), 95 (22%), and 3(1%), respectively, were treated with NPT. The indication for surgery was primary bowel perforation in 163 (37%), mesenteric ischemia in 53 (12%), anastomotic leakage in 53 (12%), ileus in 53 (12%), postoperative bowel perforation/leakage in 32 (7%), abdominal compartment in 15 (3%), pancreatic fistula in 13 (3%), gastric perforation in 13 (3%), secondary peritonitis in 11 (3%), burst abdomen in nine (2%), biliary leakage in eight (2%), and other in 15 (3%) patients. A damage control operation without reconstruction in the initial procedure was performed in 164 (37%) patients. The duration of hospital and intensive care stay were, median (range), 28 (0–278) and 4 (0–214) days. The median (range) duration of operation was 109 (22–433) min and of NPT was 3(0–33) days. A trend to shorter duration of NPT was observed over time and in the colonic perforation group. The mean operating time was shorter when only blind ends were left *in situ*, namely 110 *vs*. 133 min (*p* = 0.006). The mortality rates were 14% at 30 days, 21% at 90 days, and 31% at 1 year. An entero-atmospheric fistula was observed in five (1%) cases, most recently in 2014. Direct fascia closure was possible in 417 (95%) patients at the end of NPT, but least often (67%, *p* = 0.00) in patients with burst abdomen. During follow-up, hernia repair was observed in 52 (24%) of the surviving patients.

**Conclusion:** Open abdomen treatment with NPT is a promising concept for various abdominal emergencies, especially when treated outside normal working hours. A low rate of entero-atmospheric fistula formation and a high rate of direct fascia closure were achieved with dynamic approximation of the fascia edges. The authors recommend an early-in and early-out strategy as the prolongation of NPT by more than 1 week ends up in a frozen abdomen and does not improve abdominal sepsis.

## Introduction

Open abdomen (OA) treatment seems to be effective in treating critically ill patients with abdominal sepsis. However, the indication remains controversial as it is a resource-consuming and a non-anatomic situation with the potential of severe adverse effects (1–3). Temporary abdominal closure (TAC) with negative-pressure therapy (NPT) allows not only the patient to be resuscitated at the intensive care unit (ICU) but also the decision on the definitive surgical procedure to be postponed to a second look in an elective situation with an experienced colorectal surgeon and the aim of avoiding creation of a temporary stoma (4–6).

We initially adopted damage control surgery (DCS) for the clinical situation of perforated diverticulitis with generalized peritonitis, where we were able to report a high rate of restoration of bowel continuity in prospective studies and ultimately in a small randomized controlled trial (7–9). With the aid of dynamic sutures, we demonstrated a high rate of direct fascia closure and a low rate of hernia development (10). Simultaneously, we extended the indication for DCS with NPT to other abdominal emergencies, such as mesenteric ischemia, to allow the decision on the extent of bowel resection to be postponed to a second-look operation or to avoid stoma creation in patients with obstructed colon. After open decompression and regeneration of the overstretched colon, safe reconstruction is facilitated in a second-look operation. Moreover, in all situations outside normal working hours, and especially when working hours are subject to restrictions, the surgeon on duty can postpone the decision of performing an anastomosis DCS whenever he has doubts.

The aim of this retrospective analysis was to analyze the indications, procedures, and the outcomes of all consecutive patients treated with OA and NPT at our university hospital in the last 11 years.

## Patients and Methods

Clinical data from 438 consecutive patients treated with open abdomen at our department from January 1, 2008 to December 31, 2018 were documented in an Excel database, where data from already published prospective studies were integrated. OA treatment was indicated by the performing surgeon on duty in cases outside study protocols. Negative pressure was applied with VAC^R^ or ABThera^TM^ therapy (KCI, San Antonio, TX). To avoid fascia retraction and enhance direct fascia closure, dynamic sutures with vessel loops or approximation of the fascia edges by negative pressure was applied as published. To prevent entero-atmospheric fistula formation, direct contact between the intra-abdominal sheet of the NPT system and the intestinal sutures was avoided by covering the sides with omental fat, whenever possible. The technique to be administered for closure of the abdominal wall at the end of the OA treatment was determined by the surgeon in charge and recorded as continuous or interrupted using absorbable or non-absorbable suture material.

Statistical analysis of the data was conducted with SPSS 26.0 (Chicago, IL). Analysis was performed with the chi-square test for categorical variables or Fisher's exact test for nominal variables. Overall survival rate was calculated with a Kaplan–Meier estimate.

## Results

From January 1, 2008 to December 31, 2018, 438 patients (194 females) with a median (range) age of 66 (12–94) years, a body mass index (BMI) of 25 (14–48) kg/m^2^, and American Society of Anesthesiologists (ASA) class I–IV scores of 36 (13%), 239 (55%), 95(22%), and 3 (1%), respectively, were treated with NPT at our department.

The indication for emergency surgery is shown in detail in Table 1. Besides the main indication, primary bowel perforation with 163 (37%) and anastomotic leakage, intestinal ischemia, and ileus with 53 (12%) were the most frequent indications for the DCS procedure. Eighty-three (19%) patients suffered from a malignant disease and 21 (5%) patients were immunocompromised for solid organ transplantation. Besides, the mean ± SD operating time was 120 ± 66 min. Definitive surgery was performed in 272 (62%) and a damage control operation without reconstruction in 164 (37%) patients. In the group of patients with colonic perforation (*n* = 199), the surgical time was shorter in the damage control group, with a median (mean) time of 110 (118) min *vs*. 133 (145) min in the group where reconstruction or stoma creation was performed during the emergency surgery (*p* = 0.006). No significant difference was observed in the median (mean) durations of NPT between these two groups, namely 2 (4.3) *vs*. 3 (4.9) days.

**Table 1:** Clinical Data.

			**Female**	**Age**	**ASA Score**	**BMI**
**Indication for surgery**	***n***	**%**	**n (%)**	**Median(range)**	**Mean**	**Median(range)**
Primary bowel perforation	163	37%	71 (44%)	67 (12–92)	3.0	25 (14–40)
Anastomotic leakage	53	12%	20 (38%)	64 (36–82)	3.0	24 (14–43)
Intestinal ischemia	53	12%	21 (40%)	74 (25–95)	3.3	25 (15–37)
ILEUS	53	12%	28 (53%)	67 (21–90)	2.9	25 (14–48)
Postoperative bowel perforation	32	7%	15 (47)%	66 (26–83)	3.2	24 (15–45)
Abdominal compartment	15	3%	7 (47%)	76 (37–88)	3.4	23 (17–33)
Gastric perforation	13	3%	7 (54%)	55 (33–87)	3.3	27 (18–38)
Pancreatic fistula	13	3%	4 (31%)	60 (44–78)	3.4	26 (15–29)
Secondary peritonitis	11	3%	6 (55%9)	58 (38–77)	3.2	26 (20–33)
Burst abdomen	9	2%	7 (78%)	73 (64–80)	3.1	33 (25–40)
Biliary leakage	8	2%	2 (25%)	56 (20–84)	3.3	25 (18–44)
Other	15	3%	6 (40%)	62 (42–90)	3.3	27 (22–33)
Total	438	100%	194 (44%)	66 (12–95)	3%	25 (14–48)

*BMI, body mass index*.

**Figure 1:**
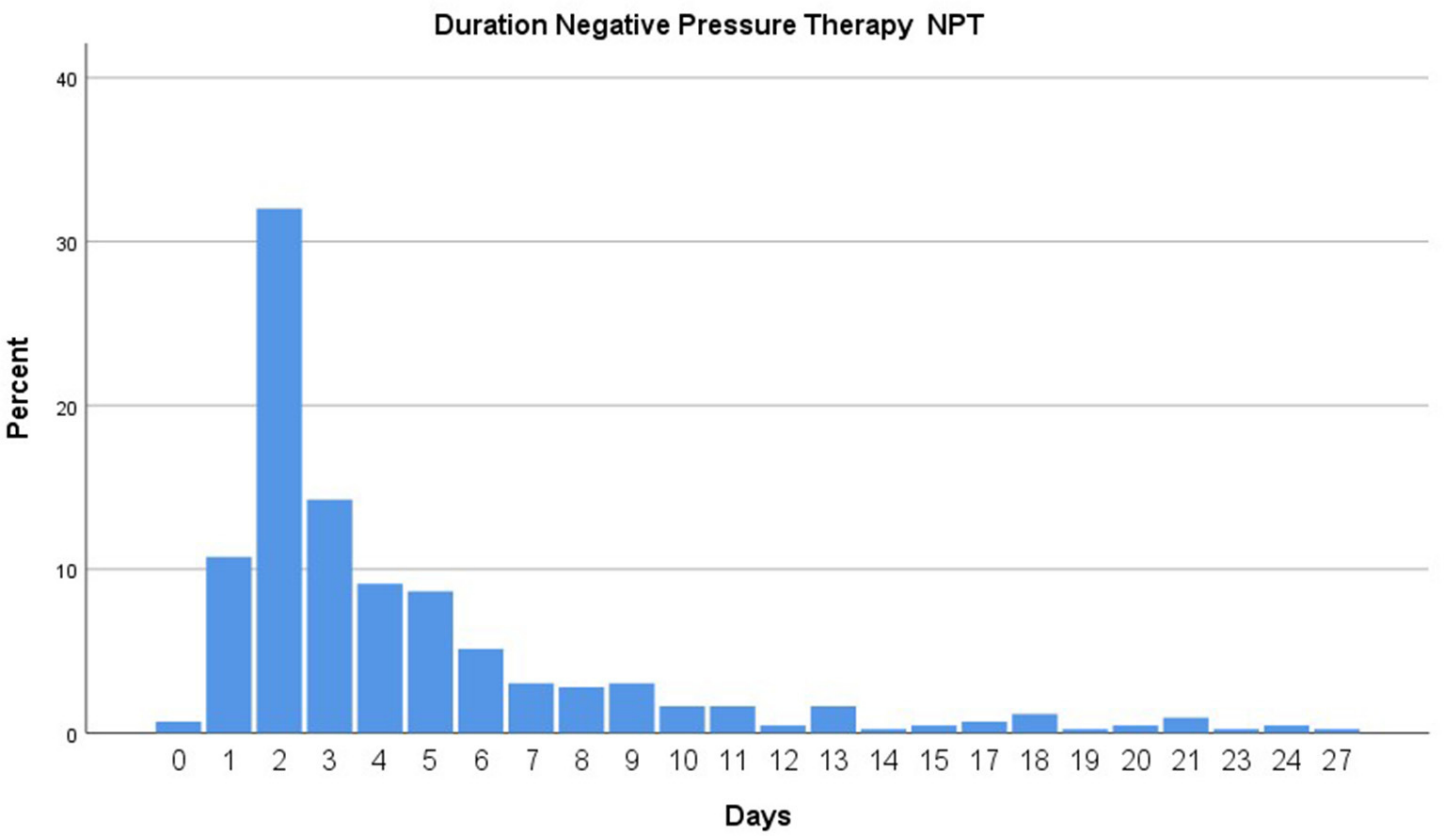
Duration of negative pressure system in place.

Outcome parameters are depicted in Table 2. The median (range) hospital stay was 28 (0–278) days, and the median (range) duration of NPT was 3 (0–27) days (see Figure 1). Admission to the ICU was not necessary in 184 patients, and the median (range) duration of ICU stay for patients admitted to the ICU (*n* = 254) was 4 (1–214) days. The mean duration of NPT was lowest in the group with intestinal ischemia, namely 3.1 days, and highest in the group with pancreatic fistula, namely 10.1 days, followed by burst abdomen (9.8 days) and biliary leakage (9.6 days, *p* = 0.027). No significant difference in ICU or hospital stay was observed between these groups.

**Table 2:** Outcome.

	**Mortality rate (%)**		**Survival rate (%)**		**Hospital stay**	**ICU stay**	**NPT**	**Rate (%)**	
**Indication for surgery**	**30-day**	**90-day**	**1 year**	**5 year**	**Median(range) days**			**Fascia closure**	**DCS**
						***n=*** **254**			
Primary bowel perforation	11%	17%	76%	66%	24 (1–257)	3 (1–61)	3 (1–28)	98%	64%
Anastomotic leakage	17%	25%	69%	60%	34.5 (3–98)	9 (1–35)	4 (1–33)	91%	28%
Intestinal ischemia	19%	26%	58%	52%	20.5 (2–128)	4 (1–51)	2 (1–21)	100%	47%
ILEUS	15%	19%	64%	54%	22 (0–176)	2.5 (1–144)	3 (2–21)	98%	21%
Postoperative bowel perforation	9%	22%	67%	56%	43 (4–278)	10 (1–170)	4.5 (1–17)	97%	9%
Abdominal compartment	20%	20%	55%	m	28 (4–56)	19 (6–34)	5 (2–18)	87%	0%
Gastric perforation	0%	15%	80%	80%	37 (13–110)	29 (16–69)	6 (1–33)	92%	8%
Pancreatic fistula	15%	23%	48%	48%	56.5 (34–192)	9 (3–61)	9 (1–21)	92%	0%
Secondary peritonitis	18%	36%	47%	47%	39 (14–112)	5.5 (1–34)	3 (1–20)	100%	0%
Burst abdomen	11%	22%	76%	31%	38 (12–72)	1 (1–214)	7 (2–24)	67%	0%
Biliary leakage	25%	25%	71%	48%	58.5 (12–140)	8 (2–64)	5 (2–27)	75%	0%
Other	13%	20%	79%	65%	23 (15–73)	5 (1–12)	2 (1–9)	93%	27%
Total	14%	21%	69%	59%	28 (0–278)	4 (1–214)	3 (1–33)	95%	38%

*ICU, intensive care unit; NPT, negative pressure therapy; DCS, Damage control surgery; m, missing*.

**Figure 2:**
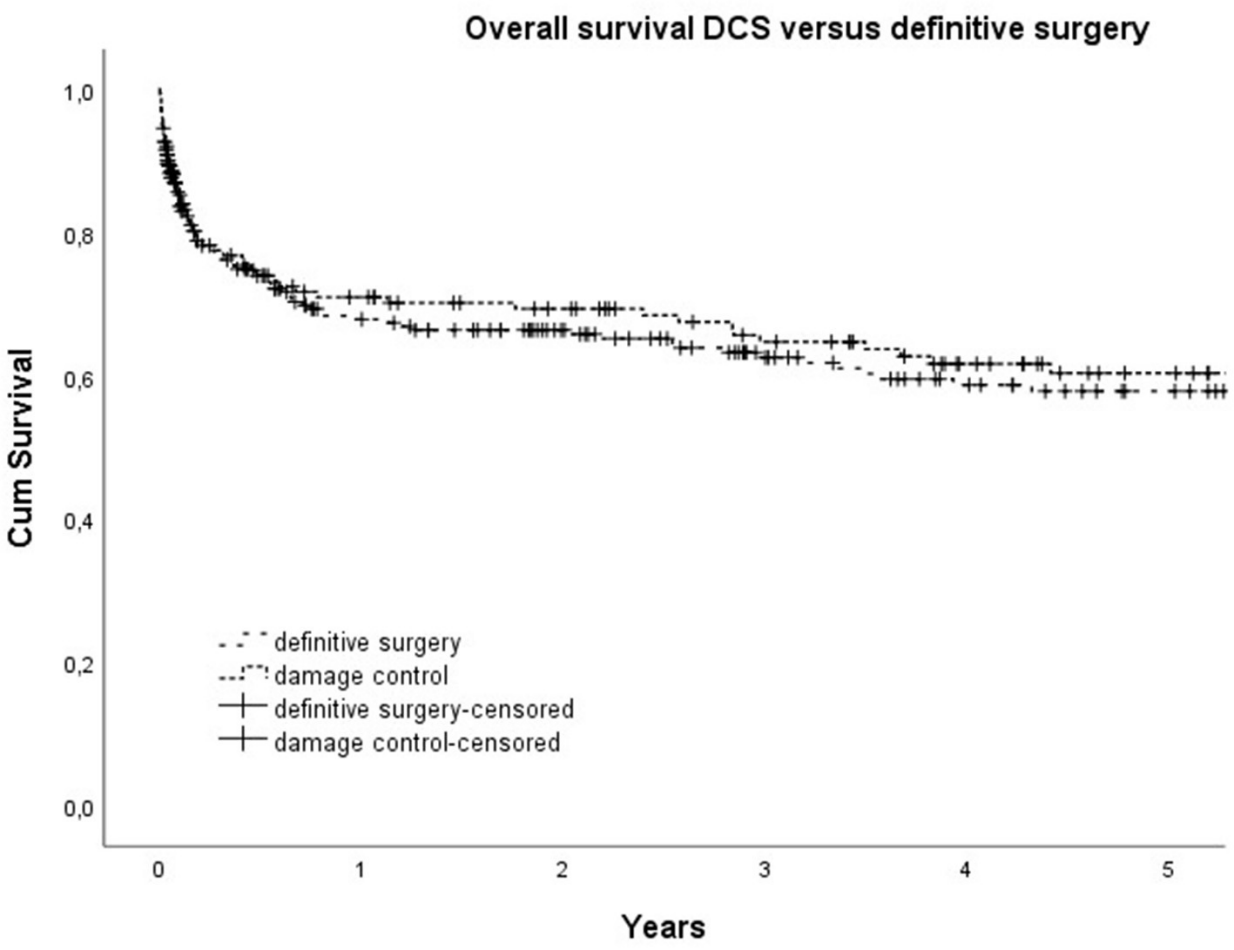
Overall survival for Damage control surgery (DCS) versus definitive surgery.

**Figure 3:**
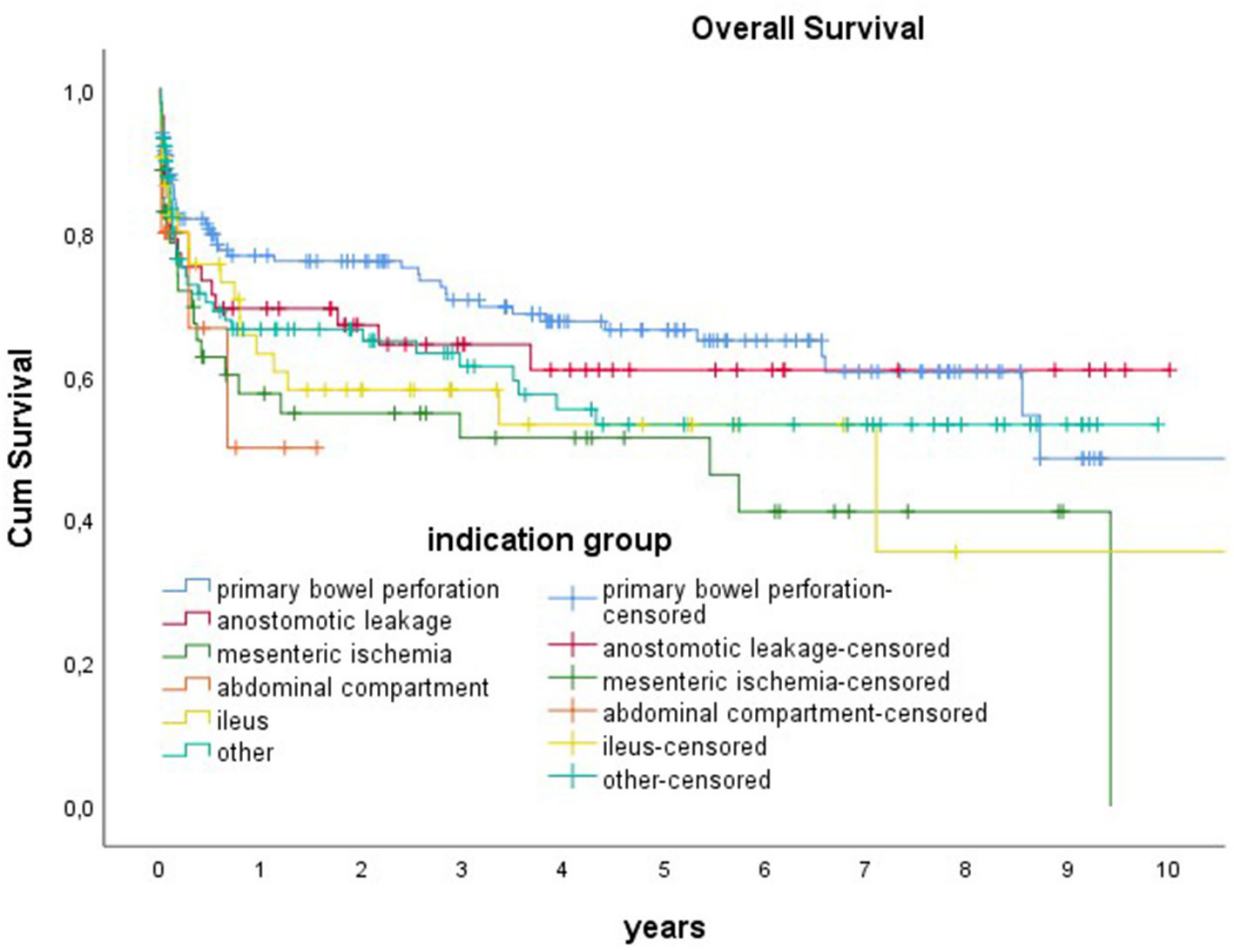
Overall survival for different indications.

The mortality rates were 14% at 30 days, 21% at 90 days, 31% at 1 year, 37% at 3 years, and 41% at 5 years. The 90-day mortality rate was highest in the group with secondary peritonitis (36%), followed by intestinal ischemia (26%), and lowest in the group with gastric perforation (15%) and primary bowel perforation (17%, n.s.) (see Figures 2, 3).

Complete closure of the fascia at the end of NPT was possible in 417 (95%) patients, nine (2%) patients died before removal of NPT, in four (1%) patients a Permacol, in two (0.5%) patients a Vicryl mesh was used to close the abdominal wall, and in four (1%) patients complete closure of the abdominal wall was not achieved. The lowest rate of direct fascia closure was observed in the group with burst abdomen (67%), followed by patients with biliary leaks (75%, *p* = 0.00). The mean (confidence interval, CI) of BMI was lowest in the patients who died before the end of NPT, with 21.9 (21.0–24.9) *vs*. 25.2 (25.0–25.5) in the group of complete fascia closure and 31.6 (29.1–34.1, *p* = 0.00) in the group of partial or mesh-mediated fascia closure.

Entero-atmospheric fistula formation as a complication of NPT was observed in five (1%) patients. Ventral hernia repair was performed in 85 patients (19% of all and 24% of all surviving patients). Body mass index was significantly higher in patients with a ventral hernia, namely a mean (95% CI) of 28.1 (27.2–28.9) *vs*. 25.0 (24.7–25.3 kg/m^2^, *p* = 0.00).

## Discussion

Damage control surgery (DCS), established in the treatment of injured patients by trauma surgeons, has been adopted by general surgeons for various abdominal emergencies (1–4). DCS meets all requirements for an emergency operation: short operating time, immediate clearance of the septic focus, and improving the patient for definitive reconstruction in a second operation at the ICU, if necessary (11). Moreover, this limited procedure can be performed by a general surgeon not specialized in colorectal surgery, a situation that is increasingly encountered especially when working hours are subject to restrictions and there is a reduced availability of specialists. The estimated risk of overtreatment appears to be tolerably low because the use of modern NPT devices means patients can be extubated and treated at the surgical ward until definitive surgery, if the patient improves rapidly after DCS (9, 11, 12).

In the setting of perforated diverticulitis with generalized peritonitis, the concept of DCS is already established (1, 9, 13). We adopted the concept also for patients with anastomotic leakage to allow reanastomosation in the second-look procedure. Of the patients treated with NPT, 53 (12%) had intestinal ischemia, where the aim was to reduce the extent of bowel resection after patient recovery or after recuperation of the intestine following thrombectomy. Another 53 (12%) patients were operated for complicated ileus. After open decompression or reposition of the incarcerated intestine, recovery of the overstretched intestinal wall allowed safe anastomosis in the second-look operation. Moreover, in cases of acute left-sided colonic obstruction due to colon cancer, DCS offers an alternative to diversion or stenting. Under elective conditions and with the aid of a colorectal surgeon, the quality of oncologic resection is enhanced.

Before the introduction of modern NPT devices, a formidable complication, namely the formation of an entero-atmospheric fistula, demanded that a strict indication be observed for OA (2). Recent studies of OA treatment with NPT report rates of entero-atmospheric fistula formation from 5 to 14%, with the risk factors of duration of OA treatment, ischemia, and cancer (14–17). In our cohort of 438 patients, where we strictly avoided direct contact between the plastic sheet and any sewn serosa lesion or an intestinal anastomosis, we demonstrate a low rate of fistula development of 1%. A further problem entailed with OA treatment, namely retraction of the fascia resulting in a ventral hernia, can be resolved with various techniques, as published in cohort studies (10, 17, 18). The technique practiced in our department, dynamic approximation of the fascia edges with vessel loops or approximation of the fascia edges with the aid of negative pressure from the beginning of NPT, resulted in a high 95% rate of direct fascia closure at the end of NPT, comparable to the data of Acosta et al. (18). The lowest rate of fascia closure, namely 67%, was achieved in the patients with burst abdomen due to septic complications or when biliary or pancreatic fistulas were observed. The need for hernia repair in 24% of the surviving patients in our cohort coincides with the published data. BMI could be identified as a risk factor (19–21).

When used as DCS, NPT was terminated in 50% of our patients after 2 days. NPT duration was longest in the group of patients with persistent pancreatic or biliary leakage and in those patients with burst abdomen due to septic complications, where conditioning of the fascia edges was awaited before the abdominal wall was definitively closed. NPT duration in abdominal sepsis is limited by the evolution of a frozen abdomen, limiting the cleansing effect of the negative pressure (22, 23). For this reason, a trend toward an earlier termination of NPT during the observation period was noted. The strategy undertaken at our department is to keep the threshold low for the indication of DCS and NPT, especially outside normal working hours, but to terminate as early as possible. Decompression laparotomy and NPT in cases of abdominal compartment gave a rare indication in 3% of our patients, while the mean duration was 6 days and fascia closure was achieved in 87% of the patients.

Nine patients died before the end of NPT. At 22, BMI was significantly lower in these patients, indicating that they had malignant or severe chronic disease. A higher BMI was a significant risk factor for complete fascia closure in our cohort of patients. The mortality rates observed in our patients appear to be comparatively low in relation to the literature (24, 25). The effects of OA and NPT on mortality and the risk of overtreatment are still up for discussion, and a prospectively randomized study was announced (12, 26, 27).

In conclusion, OA with NPT is a promising option in various abdominal emergencies, especially when used in a damage control concept and outside normal working hours, where typical and feared complications such as entero-atmospheric fistulas or fascia retraction can be successfully avoided. To demonstrate a supposed positive effect on mortality, a randomized controlled study would be helpful.

## Data Availability Statement

The raw data supporting the conclusions of this article will be made available by the authors, without undue reservation.

## Ethics Statement

Ethical review and approval was not required for the study on human participants in accordance with the local legislation and institutional requirements. Written informed consent for participation was not required for this study in accordance with the national legislation and the institutional requirements.

## Author Contributions

RK-R: design, analysis, and writing. EG: data accuisition, analysis, and writing. DR: data acquisition and analysis. JG: data acquisition. DÖ: design and data validation. AL, PG, and AP: execution and correction. All authors contributed to the article and approved the submitted version.

## Conflict of Interest

The authors declare that the research was conducted in the absence of any commercial or financial relationships that could be construed as a potential conflict of interest.

## Abbreviations

DCS: damage control surgery
OA: open abdomen
TAC: temporary abdominal closure
NPT: negative-pressure therapy
HP: Hartmann's procedure
ICU: intensive care unit
n: number
PA: primary anastomosis without ileostomy.
